# Genetic deletion of *Sphk2* confers protection against *Pseudomonas aeruginosa* mediated differential expression of genes related to virulent infection and inflammation in mouse lung

**DOI:** 10.1186/s12864-019-6367-9

**Published:** 2019-12-16

**Authors:** David L. Ebenezer, Panfeng Fu, Yashaswin Krishnan, Mark Maienschein-Cline, Hong Hu, Segun Jung, Ravi Madduri, Zarema Arbieva, Anantha Harijith, Viswanathan Natarajan

**Affiliations:** 1 Department of Pharmacology, University of Illinois, Chicago, USA; 2 Department of Bioinformatics, University of Illinois, Chicago, USA; 3 Globus, University of Chicago, Chicago, IL USA; 4 Argonne National Laboratory, Chicago, IL USA; 5 Department of Core Genomics Facility, University of Illinois, Chicago, USA; 6 Department of Pediatrics, University of Illinois, Room 3139, COMRB Building, 909, South Wolcott Avenue, Chicago, IL 60612 USA; 7 Department of Medicine, University of Illinois, Chicago, USA

**Keywords:** *Pseudomonas aeruginosa*, Pneumonia, Sphingosine kinase 2, Sphingolipids, Genomics, bacterial resistance

## Abstract

**Background:**

*Pseudomonas aeruginosa* (*PA*) is an opportunistic Gram-negative bacterium that causes serious life threatening and nosocomial infections including pneumonia. *PA* has the ability to alter host genome to facilitate its invasion, thus increasing the virulence of the organism. Sphingosine-1- phosphate (S1P), a bioactive lipid, is known to play a key role in facilitating infection. Sphingosine kinases (SPHK) 1&2 phosphorylate sphingosine to generate S1P in mammalian cells. We reported earlier that *Sphk2*^*−/−*^ mice offered significant protection against lung inflammation, compared to wild type (WT) animals. Therefore, we profiled the differential expression of genes between the protected group of *Sphk2*^*−/−*^ and the wild type controls to better understand the underlying protective mechanisms related to the *Sphk2* deletion in lung inflammatory injury. Whole transcriptome shotgun sequencing (RNA-Seq) was performed on mouse lung tissue using NextSeq 500 sequencing system.

**Results:**

Two-way analysis of variance (ANOVA) analysis was performed and differentially expressed genes following *PA* infection were identified using whole transcriptome of *Sphk2*^*−/−*^ mice and their WT counterparts. Pathway (PW) enrichment analyses of the RNA seq data identified several signaling pathways that are likely to play a crucial role in pneumonia caused by *PA* such as those involved in: 1. Immune response to *PA* infection and NF-κB signal transduction; 2. PKC signal transduction; 3. Impact on epigenetic regulation; 4. Epithelial sodium channel pathway; 5. Mucin expression; and 6. Bacterial infection related pathways.

Our genomic data suggests a potential role for SPHK2 in *PA*-induced pneumonia through elevated expression of inflammatory genes in lung tissue. Further, validation by RT-PCR on 10 differentially expressed genes showed 100% concordance in terms of vectoral changes as well as significant fold change.

**Conclusion:**

Using *Sphk2*^*−/−*^ mice and differential gene expression analysis, we have shown here that S1P/SPHK2 signaling could play a key role in promoting *PA* pneumonia. The identified genes promote inflammation and suppress others that naturally inhibit inflammation and host defense. Thus, targeting SPHK2/S1P signaling in *PA*-induced lung inflammation could serve as a potential therapy to combat *PA*-induced pneumonia.

## Background

*Pseudomonas aeruginosa* (*PA*) is an aggressive Gram-negative bacillus that causes serious opportunistic infections such as pneumonia in humans, leading to significant morbidity and mortality [1–3]. However, it is interesting to note that *PA* is also capable of causing serious infections in plants and insects with significant correlation to virulence across the species [4, 5]. Among patients, those with cystic fibrosis (CF), chronic obstructive pulmonary disease (COPD), and on mechanical ventilation are particularly prone to develop pneumonia caused by *PA* infection [6]. In fact, *PA* plays a major role in deterioration of lung function in CF patients. A highly virulent organism that can even grow in water, *PA* has of late been recognized to be capable of altering the host genome it infects in order to facilitate its own virulence [7–10]. It is known that *PA* -mediated pneumonia leads to a cascade of responses in the host, starting with innate immune response followed by increased reactive oxygen species (ROS) generation and differential regulation of sphingolipid metabolic pathways [11–13]. In the sphingolipid pathway, it has been noted that sphingosine, which is normally present in respiratory tract of healthier patients, is almost absent in CF patients [14]. On the contrary, ceramides generated by acid sphingomyelinase are known to accumulate in the airway epithelium of CF patients with pneumonia [13, 15]. Among sphingolipids, sphingosine-1-phosphate (S1P), synthesized from sphingosine by sphingosine kinases (SPHK)1 and 2, is an intercellular and intracellular bioactive lipid mediator that regulates pleotropic cellular functions under normal and pathophysiological conditions. Genetic deletion of *Sphk1*, but not *Sphk2*, in mouse caused aggravation of LPS-induced lung injury, suggesting a protective role for SPHK1/S1P signaling against endotoxemia [16]. In contrast, adenoviral overexpression of SPHK2 in wild type (WT) mouse augmented LPS-induced lung injury [16–18], while deletion of *Sphk2*, but not *Sphk1*, ameliorated *PA*-induced lung inflammation and injury in mice [11]. Using *Sphk2* knockout (KO) mice, we decided to unravel the key pathways selectively associated with SPHK2 signaling that play a role in *PA*-induced pathogenesis using differential gene expression analysis.

The infection of a host by a pathogenic microorganism initiates complex cascades of events that influence both immediate and long-term outcomes. In this study, we identified a set of *PA* responsive genes activated in the WT mice in comparison with *Sphk2*^*−/−*^. Our results show that SPHK2/S1P signaling cascade mediating *PA*-induced pneumonia modulates signaling events related to extracellular matrix remodeling, interleukin (IL) signaling, and complement cascade in the host lung. In addition, we also noted that genetic deletion of *Sphk2* resisted alteration of host pulmonary genome by *PA* infection by promoting its own virulence. The objective of this study is to identify novel pathways related to SPHK2/S1P signaling, that could contribute to the pathology as well as protection of *PA*-induced pneumonia.

## Methods

### Mouse experiments and animal care

All experiments using animals were approved by the Institutional Animal Care and Use Committee at the University of Illinois at Chicago (protocol # 15–240). *Sphk2* knockout mice were originally provided by Dr. Richard Proia (National Institutes of Health, Bethesda, MD). The knockout mice were backcrossed onto the C57BL/6 background for 8 generations. The resultant mixed background of C57BL/6 strain and the original background (F8 hybrid) was used as controls and is referred to hereafter as Wild Type (WT). All in vivo experiments were carried out with age-matched (6–8 weeks) female mice. The mice were housed in the University of Illinois Animal Care Facility. As shown in the Additional file 1 *Sphk2* showed almost complete absence of SPHK2 expression in lung tissue estimated by immunoblot of whole lung homogenates.

Anesthesia and euthanasia: The mice were anesthetized using Ketamine (100 mg/kg) and Xylazine (5 mg/kg). The animals were sacrificed and the lung tissues collected, homogenized and whole cell lysates prepared for further analysis, RNA isolation (superior lobe of right lung), and RNA-Seq studies.

### Preparation of *Pseudomonas aeruginosa* culture

The parent strain *P. aeruginosa* (*PA* 103) used for all experiments was provided by Dr. Ruxana Sadikot (Emory University, Atlanta, GA). Preparation of the cultures and determination of colony-forming units (CFU) were carried out as described previously [11, 19]. The bacterial concentration of *PA* was confirmed by plating out the diluted samples on sheep blood agar plates [11].

### Standardization of *Pseudomonas aeruginosa* inoculation and validation of bacterial load inoculated

Live PA was titrated overnight on sheep blood agar plate and PA was administered into the trachea of WT and *Sphk2*^*−/−*^ mice at a dose of 1 × 10^6^ CFU/mouse. Following administration of PA, 1.0 ml of ice-cold sterile PBS was injected into the trachea, lungs were lavaged and BAL fluid was collected, and bacterial colony count was performed at 6 or 24 h, post-inoculation by plating out the BAL samples on sheep blood agar plates.

### *Pseudomonas aeruginosa* infection of mouse lung

Age and weight-matched female WT and *Sphk2*^*−/−*^ mice were anesthetized with ketamine as per approved protocol and were administrated a single intratracheal infusion of sterile PBS or *PA*103 in PBS (1 × 10^6^ CFU/mouse). Three mice were used for each group. After 24 h of treatment, animals were euthanised; whole lung tissues were collected, and processed.

### Sample processing and RNA-Seq based gene expression analyses

Lungs were perfused with phosphate buffered saline prior to harvesting from the mice and processed immediately. Whole lung tissues were initially collected in RNA *later*® (Thermo Fischer Scientific, Waltham, MA, Cat no. AM7020) and used to isolate total RNA using microRNeasy® kit (Qiagen, Maryland, Cat no. 74004). RNA samples isolated from individual animals were separately labeled, hybridized, washed/stained and scanned according to the standard WT PLUS labeling protocol recommended by the manufacturer (Thermo Fisher Scientific, Waltham, MA).

### RNA quality control

RNA concentrations and purity were determined on a NanoDrop 1000 (Invitrogen), and RNA integrity was determined on the 2200 TapeStation system using RNA ScreenTape (Agilent, Cat. No. 5067–5576). RNA integrity number (RIN) values ranged from 7.0 to 8.4.

### RNA-Seq library preparation

Libraries were prepared with the 3′ QuantSeq mRNA-Seq Library Prep Kit REV for Illumina (Lexogen), according to manufacturer’s instructions. In brief, 10–500 μg of total RNA was used to make each library. Library generation was initiated by oligo (dT) priming followed by first strand cDNA synthesis, removal of RNA and second strand cDNA synthesis using random priming and DNA polymerase. During these steps Illumina linker sequences and external barcodes were incorporated. Next the libraries were subject to final 20 cycles of PCR amplification.

### RNA-Seq library validation and quantification

Quality of the libraries was checked on the 2200 Tape Station system using D1000 ScreenTape (Agilent, Cat. No. 5067–5582), and as expected, peaks ranged from 264 to 294 bp. Libraries were quantified on the Qubit 2.0 Fluorometer with the Qubit dsDNA HS Assay Kit (Life Technologies, Cat. No. Q32854). Individual libraries were pooled in equimolar amounts and concentration of the final pool was determined by PCR quantification method using KAPA Library Quantification Kit (KAPA Biosystems). Sequencing was carried out on NextSeq 500 (Illumina), 1 × 75 nt reads, high output, to achieve approximately 20 × 10^6^ clusters per sample.

Genomics Suite 6.6 statistical package (Partek, Inc., Saint Louis, MO) was used to process hybridization signals collected. The parameters applied for hybridization signal processing were as follows: RMA algorithm-based background correction, quantile normalization procedure, and probe set summarization [20, 21].

All processed array files were inspected for the quality metrics such as average signal present, signal intensity of species-specific housekeeping genes, relative signal intensities of labeling controls, absolute signal intensities of hybridization controls, and across-array signal distribution plots [22]. All hybridizations passed quality control according to indicated labeling and hybridization controls.

### Identification of differentially expressed transcripts

In order to identify the subset of genes modulated specifically to the infection of WT and *Sphk2*^*−/−*^ mice, we performed a two-way ANOVA using the status of *PA* infection and *Sphk2* expression as comparison factors. We compared the following groups: *Sphk2*^*−/−*^ *PA* infected (*Sphk2*^*−/−*^ *PA*), *Sphk2*^*−/−*^ control (*Sphk2*^*−/−*^ CTRL), Wild Type *PA* infected (WT *PA*) and Wild Type control (WT CTRL). ANOVA model was based on Method of Moments [23] in combination with Fisher’s Least Significant Difference (LSD) contrast (Tamhane and Dunlop, 2000). The Fisher’s contrast allowed calculation of direction and magnitude of change for all pair-wise comparisons between the treatment groups and was later validated by RT-PCR. Raw reads were aligned to reference genome using Burroughs-Wheeler Aligner Maximal Exact Matches (BWA-MEM) [24]. Gene expression was quantified using FeatureCounts [25]. Differential expression statistics (fold-change and *p*-value) were computed using edgeR [26, 27], generalized linear models to model the effect of genotype, infection, and their interaction. We used Globus Genomics [28] for these analyses. Calculated raw *p*-values were adjusted for False Discovery Rate (FDR) according to Benjamini-Hochberg (BH) correction procedure [29, 30]. Significant genes were determined based on an FDR threshold of 5% (0.05) and plotted in a heatmap. The FDR incorporates sample size in each group, sequencing depth and gene expression variability. The significance calculated is an output dependent on these factors. In spite of reducing the number in one group to two and comparing with the three in other groups, the data show significant changes in number of genes as shown in the results with FDR set at 0.05. The data and the level of significance presented are independent of human error. Pathway enrichment analysis on differentially expressed genes was performed using the Pathway Maps database in MetaCore. The top 35 genes, based on the interaction term FDR, were plotted in a heatmap. Additionally, we compared the significantly differentially expressed (FDR < 0.05) genes based on genotype, infection, or their interaction in a Venn diagram.

### Availability of data and materials

The RNA-Seq datasets supporting the conclusions of this article are available in the National Center for Biotechnology Information Gene Expression Omnibus repository, with unique persistent identifier of NCBI tracking system accession number. The accession number is GSE12359. The hyperlink to the datasets is given below.

https://www.ncbi.nlm.nih.gov/geo/query/acc.cgi?acc=GSE121359

### Pathway enrichment analyses and data visualization

We performed pathway enrichment analyses (EA) in order to identify the biological factors driving the protective effect observed in *Sphk2*^*−/−*^ mice with *PA* pneumonia. Transcripts identified as differentially expressed in KO animals in response to *PA* infection in two-way ANOVA test (FDR cut off of 0.05) were imported into MetaCore Genomic Analyses Tool Release 6.22 (Thomson Reuters) for analyses.

**Fig. 1:**
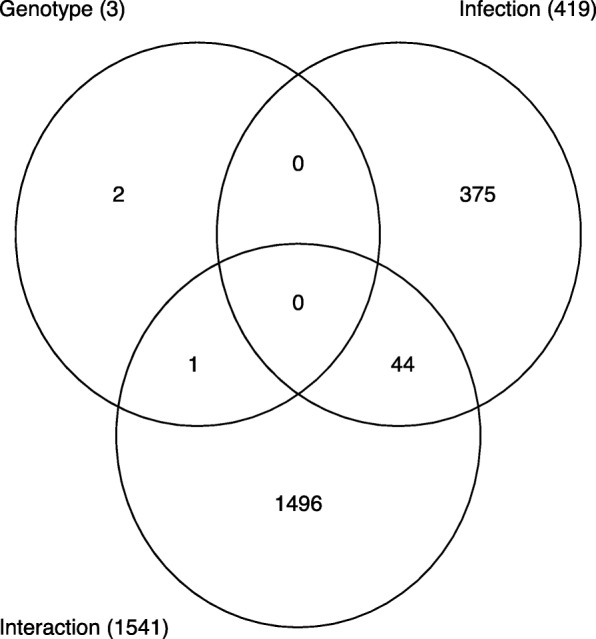
The number of genes differentially regulated in the WT and Sphk2−/− mice exposed to PA shown as a Venn diagram. Data was analyzed using two-way ANOVA. Two-way ANOVA represents the analyses performed to ascertain the impact of three factors such as 1. *Sphk2* gene KO, 2.*PA* infection, 3. Interaction of the gene KO and infection There are three circles representing the data derived from the two-way ANOVA. Circle labelled genotype shows genes affected by knockout of *Sphk2* gene. *Sphk2*^*−/−*^ shows only 2 genes differentially regulated when the corresponding gene was knocked out. Circle labelled infection shows genes affected by *PA*. 375 genes differentially regulated by *PA* group compared to the corresponding control unaffected by other factors. The third circle shows genes affected by the interaction between the two factors ie *Sphk2*^*−/−*^ and *PA*. 1496 genes were solely affected by the interaction between *Sphk2*^*−/−*^ and *PA*

**Fig. 2:**
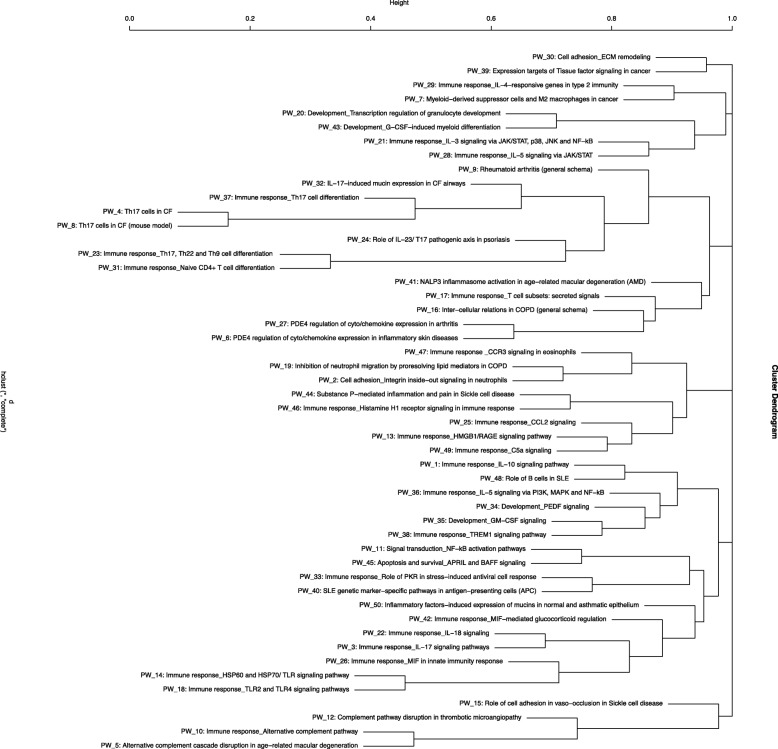
Cluster dendrogram showing differentially regulated nodal biological pathways in the animal model of *PA*-induced pneumonia. WT and *Sphk2*^*−/−*^ mice were exposed to *PA* for 24 h in our animal model of *PA* pneumonia. Lung tissues isolated at the end of treatment were investigated as described in the Material and Methods. In order to delineate the underlying biological events that could be related to the protective effect seen in *Sphk2*^−/−^ mice against *PA* pneumonia, pathway enrichment analysis was performed. The 6 M clusters of pathways were identified and grouped by similar functions, thus highlighting biological motifs most prevalent in our model are shown in here

**Fig. 3:**
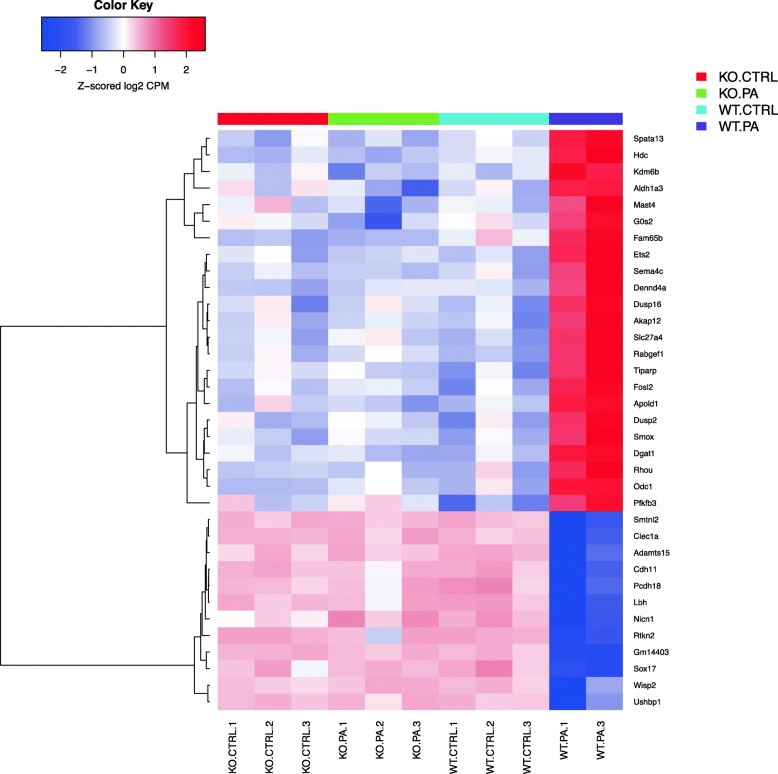
Heatmap showing genes identified as maximally differentially regulated in the animal model of *PA* pneumonia. This heatmap depicts the top 35 differentially expressed genes between the four groups: WT Control (WT CTRL), WT PA infection (WT PA), *Sphk2*^*−/−*^ control (*Sphk2*^*−/−*^ CTRL), *Sphk2*^*−/−*^ PA infection (*Sphk2*^*−/−*^ *PA*)*.* “Pathway Maps” ontology was used to analyze differentially expressed genes. 50 most enriched pathways (PW) were identified, and nodal PWs were clustered based on their gene content with stress on reduced duplication. Initially, a complete linkage hierarchical clustering on the Jaccard distance between the complete set of genes in each PW was done. This was followed by identification of closely related individual entities. Using a dissimilarity cut off of 0.6, each cluster of closely related PWs was taken as one mega pathway. Heatmaps were created by combing the associated differential genes in order to analyze gene interactions. Details of clustering pathways has been shown in Table 1. The color key shows the z-scored normalized expression level ranging from dark blue to dark red. The corresponding degree of differential regulation ranges from − 2 of down regulation or more to + 2 of upregulation or more

**Fig. 4:**
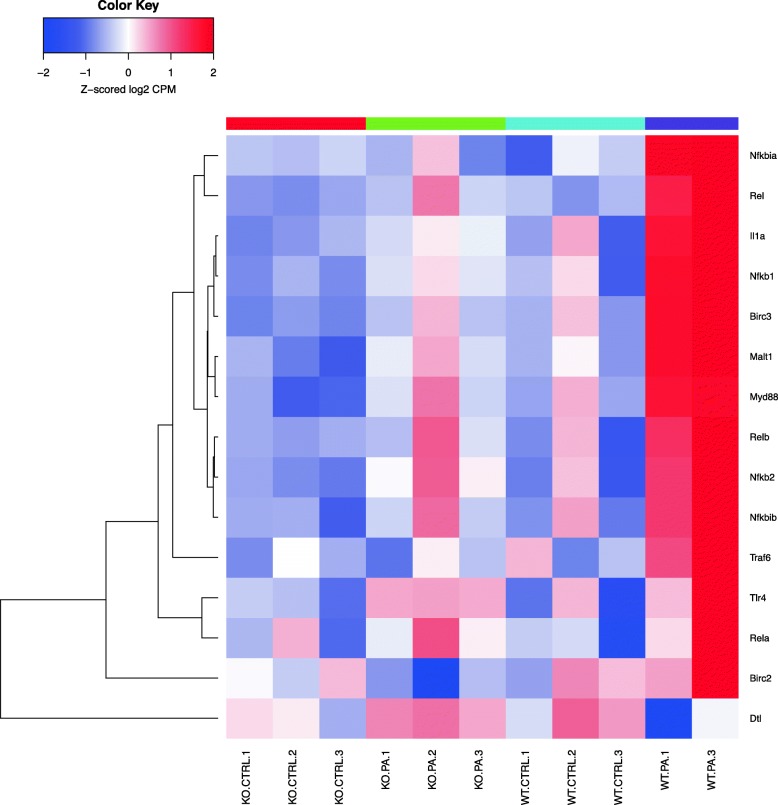
Genes differentially regulated in the immune response following *PA* infection (cluster 1) and NF-κB. This heat map shows the biological nodal pathway related to immune response showing differential regulation of genes among the 4 different groups as described. Key genes seen in the heat map are described here. A significant upregulation of genes such as *Rela, Tlr4, Traf6, Nfkbib, Nfkb2, Relb, Nfkb1, Rel* was observed

**Fig. 5:**
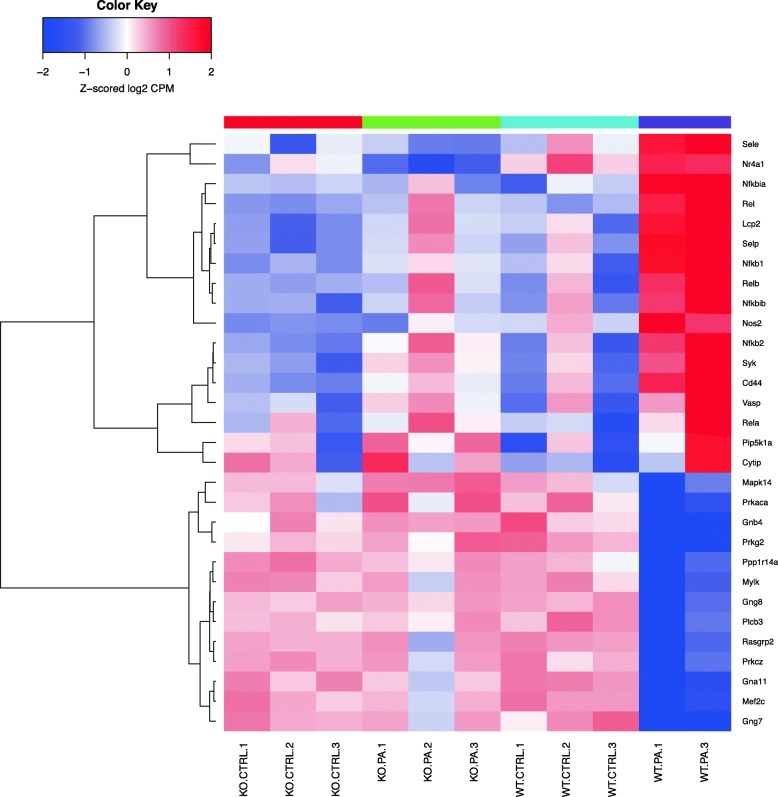
Genes differentially regulated in the nodal pathway related to PKC δ (cluster 8). This heatmap cluster combines data from related pathways shown in Additional file 3: Table S2. Among the genes prominently up-regulated by *PA* in the WT include *Sele, Pip5k1a, Lcp2, Nr4a1, Selp and Nos2*. The genes down-regulated include *Gng7, Mef2c, Gna11, Prkcz, Rasgrp2, Ppp1r14a, Prkg2, Gnb4 and Prkaca*

**Fig. 6:**
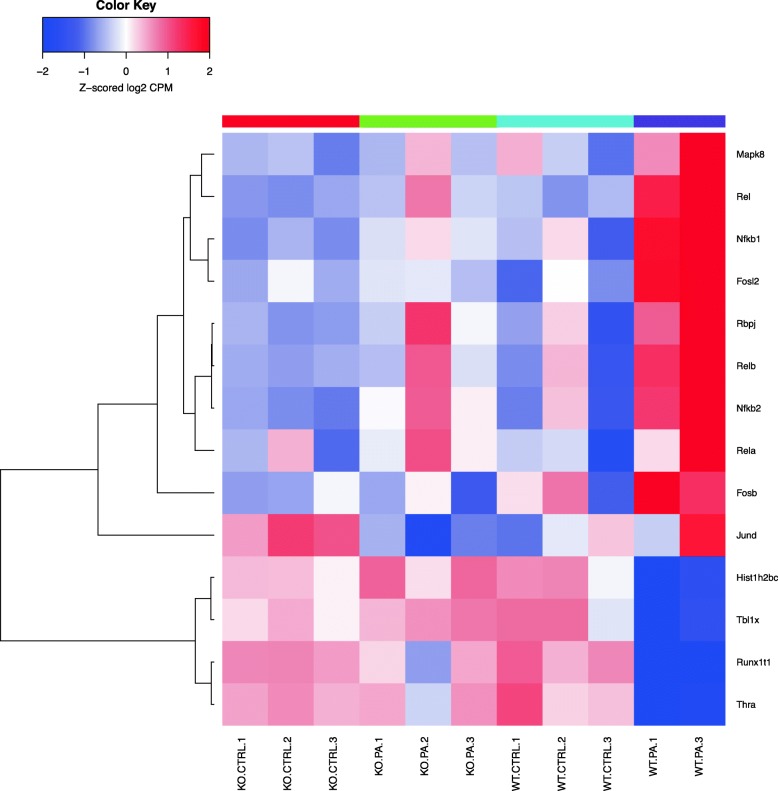
Genes differentially regulated in the nodal pathway related to epigenetic regulation (cluster 10). Selected genes depicted in the heatmap are described here. Heatmaps were prepared based on clustering of closely related pathways. Heatmaps show overlap of genes as there is overlap of genes among related pathways. The genes down-regulated are *Thra, Runx1t1, Tbl1x, Hist1h2bc. Significant genes upregulated are Jund, Fosb, Rbpj, Fosl2*

**Fig. 7:**
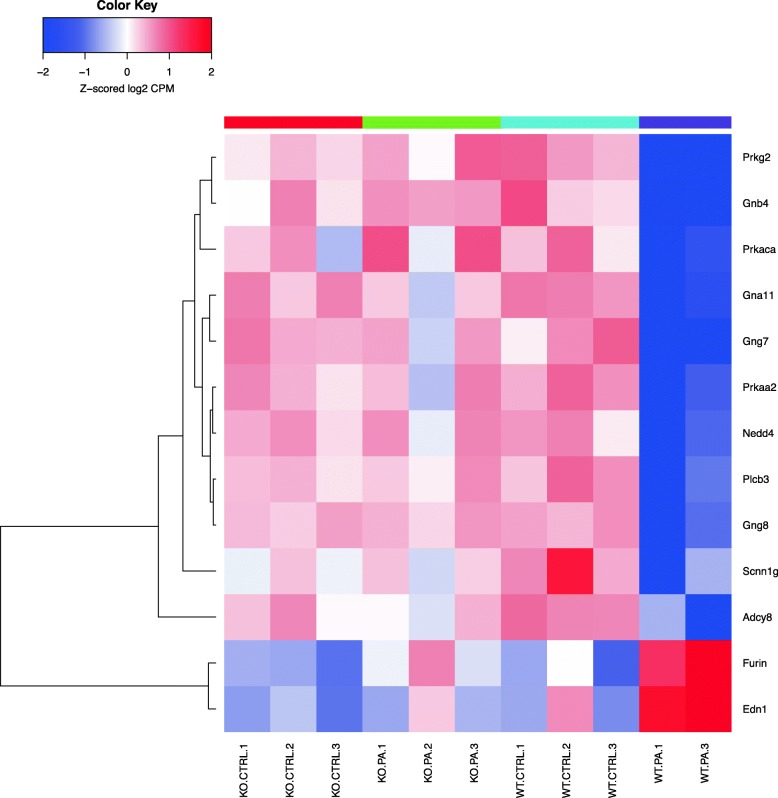
Genes that are differentially regulated in the nodal pathway related to cluster 11a. This cluster details the Epithelial sodium channel regulation in normal and cystic fibrosis airways. Among the genes downregulated in WT *PA* in contrast to *Sphk2*^*−/−*^ *PA* are those coding for *Adcy8*- the adenylate cyclase 8 gene, *Scnn1g*- the gamma subunit of sodium channel, *Plcb3, Nedd4. Furin and Edn1* were up-regulated in WT PA compared to *Sphk2*^*−/−*^ PA. *Edn1* belongs to endothelin family

**Fig. 8:**
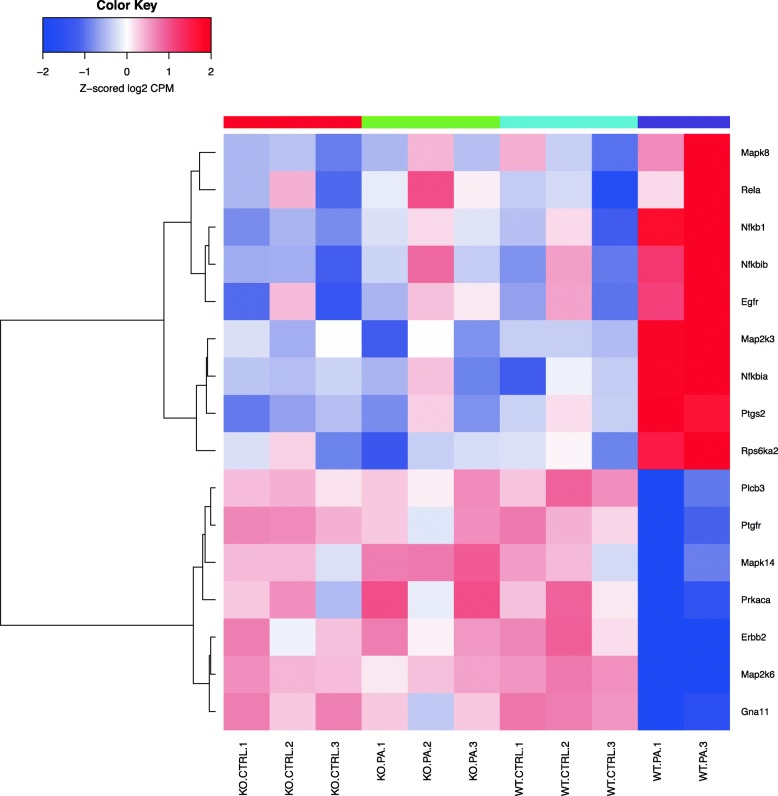
Genes that are differentially regulated in the nodal pathway related to mucin expression (cluster 11 b). *Rps6ka2, Ptgs2, Nfkbia, Egfr* genes are shown as significantly up-regulated in WT *PA* compared to the rest. Genes for *Gna11, Map2k6, Erbb2, Mapk14* and *Ptgfr* are down-regulated in WT *PA* compared to *Sphk2*^*−/−*^ *PA* and the rest of groups

**Fig. 9:**
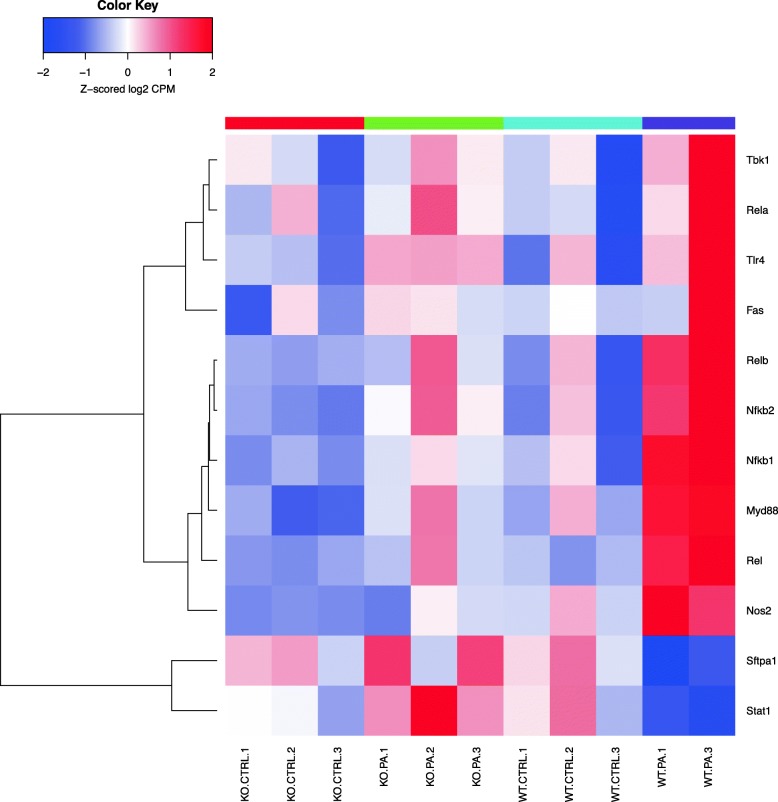
Genes that are differentially regulated in the nodal pathway related to bacterial infection (cluster 12). The following genes related to bacterial infection were down-regulated in WT *PA*: *Nos2, Rel, Myd88, Nfkb1, Nfkb2, Relb, Fas* and *Tlr4*. An overlap with NF-κB pathway is noted here. Down regulated genes include *Stat1 and Sftpa1*

**Fig. 10:**
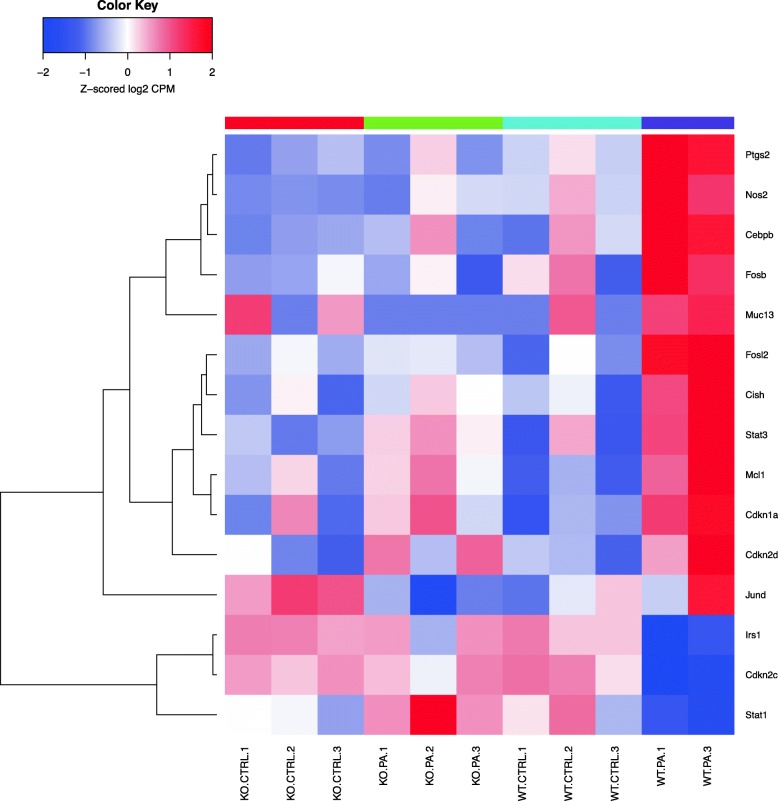
Genes that are differentially regulated in the nodal pathway related to IL-6. The following genes were among the up-regulated ones in the WT *PA* group compared to the rest *Cdkn2d, Cdkn1a, Mcl1, Stat3, Cish* and *Fosl2*. Notably, *Cish* represents CIS family members which are known to be cytokine-inducible negative regulators of cytokine signaling. The down regulated genes include *Stat1, Cdkn2c and Irs1*

Differentially expressed genes were analyzed using the “Pathway Maps” ontology and the top 50 most enriched pathways (PW) were identified. The output of analyses using the tool contained a substantial number of individual PWs that overlap by genes, representing the sub-segment of the same PWs and creating redundancy. In order to reduce duplication, we clustered nodal PWs based on their gene content in order to reduce duplication. Complete linkage hierarchical clustering on the Jaccard distance between the complete set of genes in each PW was used to identify closely related individual entities. A measure of the dissimilarity between two PWs (based on their gene sets) with scales from 0 to 1 was used; ‘0’ if the sets are exactly the same, and ‘1’ if they are completely different and have no genes in common. For the purpose of biological interpretations, we considered each cluster of closely related PWs as one unit or mega pathway (dissimilarity cut off of 0.6). We combined all associated differential genes for analyzing gene interactions and creating heatmaps as shown in the Venn diagram (Fig. 1) and a dendrogram (Fig. 2). The heatmaps for selected mega pathways were created by plotting z-scored normalized expression levels of differentially expressed genes (FDR < 0.05) across all experimental groups (Figs. 3, 4, 5, 6, 7, 8, 9 and 10). The z-scored normalized expression level using the color key ranging from dark blue to dark red.

### Realtime RT- PCR validation of RNA-Seq results

Total RNA was isolated from mouse lung homogenate using TRIzol® reagent according to the manufacturer’s instructions and purified using the RNeasy® Mini Kit according to the manufacturer’s protocol (Qiagen, MD, USA). Quantitative RT-PCR was done using iQ SYBR Green Supermix using iCycler by Bio-Rad, USA. 18S rRNA (sense, 5′-GTAACCCGTTGAACCCCATT-3′, and antisense, 5′- CCATCCAATCGGTAGTAGCG-3′) was used as external control to normalize expression [31]. All primers were designed by inspection of the genes of interest using data from PrimerBank database (Harvard University, Boston USA). The sequence description of mouse primers used are given in Additional file 2: Table S1. Negative controls, consisting of reaction mixtures containing all components but the target RNA, were included with each of the RT-PCR runs. The representative PCR mixtures for each gene were run in the absence of the RT enzyme after first being cycled to 95 °C for 15 min in order to ensure that amplified products did not represent genomic DNA contamination. No PCR products were observed in the absence of reverse transcription. Direct comparison of four groups such as WT control, WT *PA*, *Sphk2*^*−/−*^ control and *Sphk2*^*−/−*^ *PA* was done using ANOVA test, as described earlier. The level of statistical significance was set at *p* < 0.05.

Validation studies were performed in more animals in addition to the cohort used in RNA-Seq studies.

## Results

### Shared and differentially expressed genes in wild type and *Sphk2*^*−/−*^ mouse lungs with or without *PA* exposure

Analysis of gene expression showed that 375 genes were differentially regulated by *PA* infection of mouse lungs compared to the corresponding uninfected control mice. Venn diagram showing the number of genes differentially regulated in the WT and *Sphk2*^*−/−*^ mice exposed to *PA* based on two-way ANOVA analysis is shown in Fig. 1. under three different categories viz.: 1. *Sphk2* gene knockout, 2. Exposure of the mouse *to PA*, and 3. Interaction of *Sphk2* gene knock out and *PA*. The intersecting areas show the number of genes affected by the corresponding condition. The advantage with two-way ANOVA is that the third variable of interaction between the two factors is purely dependent on interaction, thus independent of the direct effect of the other two variables. Only 2 of the 375 differentially expressed genes (DEGs) could be strictly characterized as those affected solely due to the impact of genetic deletion of *Sphk2* (Fig. 1). It is also interesting to note that 1496 genes were affected by the interaction between deletion of *Sphk2* and *PA* infection of the mouse lung.

### Pathway enrichment (PW) analyses revealing underlying biological currents

The top 50 differentially regulated pathways were identified based on the gene expression profiles and relationship between the PWs is demonstrated in Fig. 2. A list of the identified fifty PWs in the order of their position on hierarchical clustering graph and details of the clustering of similar pathways are provided in Additional file 3: Table S2. As described in the methods, a dissimilarity score of 0.6 was used as cut off for the selection of clustering pathways and reducing the redundancy. The data were condensed to eight cluster PWs that are differentially regulated among the four groups (WT CTRL, WT *PA*, *Sphk2*^*−/−*^ CTRL and *Sphk2*^*−/−*^ *PA*) studied, and heatmaps show differential expression of selected genes (Figs. 3, 4, 5, 6, 7, 8, 9 and 10). The first heatmap provides a depiction of the top 35 differentially expressed genes between the groups (Fig. 3). The following are the most significant PW clusters: 1. Immune response to *PA* infection (cluster 1) and NF-κB signal transduction (cluster 3), 2. PKC signal transduction (cluster 8), 3. Impact on epigenetic regulation (cluster 10), 4. Epithelial sodium channel pathway (cluster 11a), 5. Mucin expression (cluster 11b), and 6. Bacterial infection related pathway (cluster 12) (Figs. 4, 5, 6, 7, 8 and 9).

### Differentially regulated genes in the PW clusters

WT mice challenged with *PA* showed significant upregulation of the genes related to NF-κB pathway in contrast to similarly treated *Sphk2*^*−/−*^ mice (Fig. 4). Of the five genes encoding for the *Nfκb* family, there was significant increase in expression of NF-κB1, NF-κB2, Rel A and Rel B. Genes encoding for NF-κB inhibitors alpha and beta were also significantly elevated in the WT *PA* group compared to the rest of the groups. Interestingly, the *Dtl* gene, which encodes for TNF superfamily member 13b (TNFRSF13B), was down-regulated in the WT *PA* compared to the rest of the groups. This cytokine expressed in B cell lineage acts as a potent B cell activator stimulating their proliferation and differentiation [32].

Recently, a novel role of Protein Kinase C-δ (PKC-δ) in *PA*-induced phosphorylation of SPHK2 and histone acetylation was demonstrated in lung epithelial cells [11]. In this context, we noted that genes in the PKC pathway have undergone significant differential regulation following infection with *PA* of both WT and *Sphk2*^*−/−*^groups. PKC family of proteins activate target proteins by promoting phosphorylation at serine and threonine amino acid residues [33]. In the WT *PA* group upregulation of virulence promoting members of the PKC family such as *Sele* (stimulating adhesion of leucocytes) [34], and *Lcp2* and *Nr4a1* that promote apoptosis [35] was observed. Further, a significant down-regulation of genes essential for maintenance of normal metabolic state in the WT *PA* group belonging to the PKC family was observed. The down-regulated genes included *Prkcz, Prkaca, Prkg2, Ppp1r14a and Plcb3.* These genes were maintained in an up-regulated state in the control as well as the *Sphk2*^*−/−*^ group challenged with *PA*. *Prkcz* is a member of the PKC family, which, unlike the classical PKC isoenzymes, shows calcium and diacylglycerol (DAG) independent activation. *Prkaca*, protein kinase A catalytic subunit (PKA Cα) is a member of the AGC kinase family, contributing to the control of cellular processes such as glucose metabolism and cell division [36]. *Prkg2* encodes Protein Kinase, CGMP-Dependent, Type II, is a crucial regulator of intestinal secretion and bone growth [37, 38]. This protein also phosphorylates and activates CFTR on the plasma membrane. *Ppp1r14a*, protein phosphatase 1 regulatory subunit 14A, is an inhibitor of smooth muscle myosin phosphatase. *Plcb3* encodes phospholipase C beta 3 that catalyzes the production of the second messengers such as DAG and inositol 1,4,5-triphosphate from phosphatidylinositol via G-protein-linked receptor-mediated signal transduction.

Genes affecting epigenetic pathways were also noted to be differentially regulated. *PA* infection causes downregulation of Hist1h2bc and *Runx1t1* genes. While the former gene represents the histone cluster 1, *H2bc*, the latter represents *Runx1* translocation partner 1. This gene encodes a member of the myeloid translocation gene family that interacts with DNA-bound transcription factors leading to recruitment of a range of corepressors thus causing transcriptional repression [39].

Further, we noted differential regulation of genes coding epithelial sodium channels (ENaC) in vertebrates. *Scnn1g* gene coding for the γ subunit of the ENaC was significantly down-regulated in WT *PA* but remained up-regulated in the rest of groups. WT *PA* was associated with up-regulation of *Furin* gene that was not clearly observed in *Sphk2*^*−/−*^ mouse lungs. *Furin* is a host cell factor that significantly enhances virulence of viral infection in cultured cells [40]. In the mucin expression pathway, Erb-B2 Receptor Tyrosine Kinase 2 gene encodes a member of the epidermal growth factor (EGF) receptor family of receptor tyrosine kinases, which was down- regulated in the WT *PA* group. Following identification of the PWs, we validated differential expression of specific genes by their biological impact, and further validated using real time RT-PCR.

We would like to describe the two genes that were changed between the KO and the WT in absence of infection. The genes were Frs3 (Fibroblast growth factor receptor substrate 3) and Zbtb16 (Zinc finger and BTB domain containing 16).

Frs3 gene encodes a substrate for the fibroblast growth factor receptor [41]. The encoded protein is present in the plasma membrane and links fibroblast growth factor receptor stimulation to activators of Ras [42]. This follows down-regulation of extracellular regulated kinase 2 through direct binding [43].

Zbtb16 gene is a member of the Krüppel-like family of transcription factors (KLFs) which comes under the C2H2-type zinc-finger protein family [44, 45]. Zbtb16 gene specifically encodes a zinc finger transcription factor that contains nine Kruppel-type zinc finger domains at the carboxyl terminus [46]. Located in the nucleus, similar to Sphingosine kinase 2, this protein is involved in cell cycle progression, and interacts with histone deacetylase [47, 48].

The top 50 differentially expressed genes are summarized in Additional file 3: Table S2.

### Realtime RT- PCR validation of RNA-Seq results

**Fig. 11:**
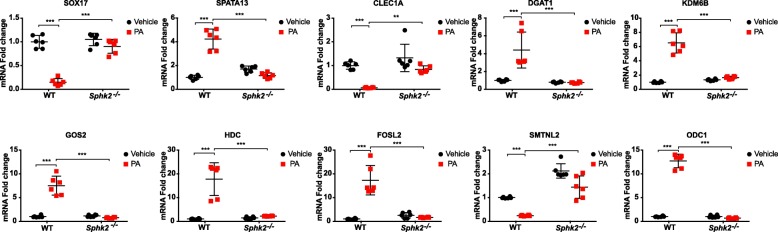
Validation by RT-PCT of differentially regulated genes in microarray. The figures represent RT-PCR results in red and black solid squares. Validation was done using the most differentially expressed genes from the gene pool identified by the microarray. The genes studied were *Spata13, Hdc, G0 s2, Fosl2, Dgat1, Kdm6b, Odc1, Smtnl2, Clec1a, and Sox17*

Table 1 and Fig. 11a & b show real-time RT-PCR performed on select 10 genes based on the observations made from the RNA-Seq gene expression analysis. RT-PCR confirmed all the 10 genes suggesting 100% validation in terms of both vectoral changes and significance in ANOVA test.

**Table 1:** A description of the function of the genes used for validation of RNAseq data

Name of gene	Function of the corresponding protein
Spata13 Spermatogenesis Associated 13	Serves as guanine nucleotide exchange factor (GEF) for RHOA, RAC1 and CDC42 GTPases. Involved in regulation of cell migration and adhesion assembly and disassembly.
Hdc Histidine Decarboxylase	Converts L-histidine to histamine
Kdm6b Lysine Demethylase 6B	Lysine-specific demethylase which specifically demethylates di- or tri-methylated lysine 27 of histone H3
G0 s2 G0/G1 Switch 2	Promotes apoptosis by binding to BCL2, thus preventing the formation of anti-apoptotic BCL2-BAX heterodimers
Fosl2 FOS Like 2, AP-1 Transcription Factor Subunit	The gene encodes leucine zipper proteins which can dimerize with proteins of the JUN family. Resulting in formation of the transcription factor complex AP-1. FOS proteins act as regulators of cell proliferation, differentiation, and transformation.
Dgat1 Diacylglycerol O-Acyltransferase 1	Catalyzes conversion of diacylglycerol and fatty acyl CoA to triacylglycerol
Odc1 Ornithine Decarboxylase 1	Catalyzes the first and rate-limiting step of polyamine biosynthesis converting ornithine into putrescine. Putrescine is the precursor for the polyamines which are essential for cellular processes, ranging from DNA replication to apoptosis.
Smtnl2 Smoothelin Like 2	Smoothelin is a structural protein found exclusively in contractile smooth muscle cells
Clec1a C-Type Lectin Domain Family 1 Member A	This protein serves diverse functions, such as cell adhesion, cell-cell signaling, inflammation and immune response
Sox17 Sex Determining Region Y Box 17	Is a transcription regulator binding to target promoter DNA and bends the DNA.

The genes identified as maximally differentially regulated are represented in the heatmap shown in Fig. 3. The genes for RT-PCR validation were selected from those maximally differentially regulated genes and identified as significant from the various differentially regulated nodal pathways as they were related to cell migration, inflammation, epigenetic regulation of genome, and IL-6 signaling. *Spata13, Hdc, Kdm6b, Gos2, Fosl2, Dgat1, Odc1, Smtnl2, Clec1a* and *Sox17* were therefore chosen for RT-PCR validation. In addition, these genes were also related to biologically relevant findings in our animal model.

### Western blot validation of RNA-Seq results

**Fig. 12:**
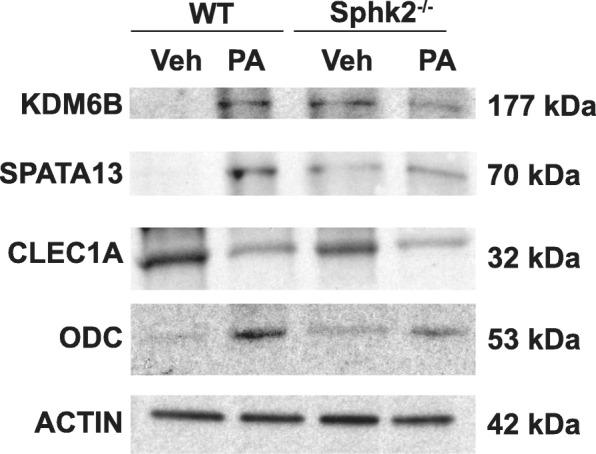
Validation by Western Blot of differentially regulated genes in microarray. WT or *Sphk2*^*−/−*^ mice were treated with *Pseudomonas aeruginosa* (*PA*) or vehicle (Veh) for 24 h following which the mice were euthanized, lungs removed for protein and RNA extraction as described in Materials and Methods. Whole lung homogenates were subjected to SDS-PAGE and Western blotting . Immunoblot showed increased expression of JmjD3, Spata 13 and ODC1 in the *PA* exposed lungs of the WT mice compared to *Sphk2*^*−/−*^ mice. Clec1A expression was significantly decreased in *Sphk2*^*−/−*^ exposed to *PA*, compared to WT *PA* infected mouse lung. Western blots probed with appropriate primary and secondary antibodies were quantified by densitometry and normalized to the corresponding total protein

Western blot analyses were performed to validate protein expression of genes that were modulated in for Real-time RT-PCR (Fig. 12). Immunoblots showed increased expression of KDM6B, SPATA13 and ODC1 in the *PA* exposed lungs of the WT mice compared to *Sphk2*^*−/−*^ mouse lungs. CLEC1A expression was significantly decreased in *Sphk2*^*−/−*^ mice exposed to *PA*, compared to WT *PA*. Western blots probed with corresponding antibodies were quantified by densitometry and normalized to the corresponding total protein. This finding correlated well with the results of the RNA-Seq and real-time RT-PCR analysis.

### Biological impact of *Sphk2* deletion in the animal model correlates with the differential expression of genes in mouse lungs

Our recently published data from the animal model showed that deletion of *Sphk2*, but not *Sphk1*, protected mice against *PA-*mediated inflammatory lung injury [11]. Following intratracheal instillation of *PA* (1 × 10^6^ CFU/animal), infiltration of PMNs in the lungs and increased protein levels in BALF were noted in WT mice, whereas these responses were significantly blunted in *Sphk2*^*−/−*^ mice. Following *PA* infection, BALF concentrations of the pro-inflammatory mediators IL-6 and TNF-α were significantly elevated in WT compared to *Sphk2*^*−/−*^ mice. Further, infection of mouse lungs with *PA* revealed enhanced phosphorylation of PKC δ and phospho-SPHK2 immunostaining predominantly in the nucleus of lung epithelial cells, and inhibiting PKC δ or SPHK2 activity with small molecule inhibitor attenuated *PA*-induced H3 and H4 histone acetylation and IL-6 secretion in lung epithelial cells. These in vivo and in vitro results confirm the PW cluster analysis.

### Validation of equal bacterial load inoculated into each mouse

**Fig. 13:**
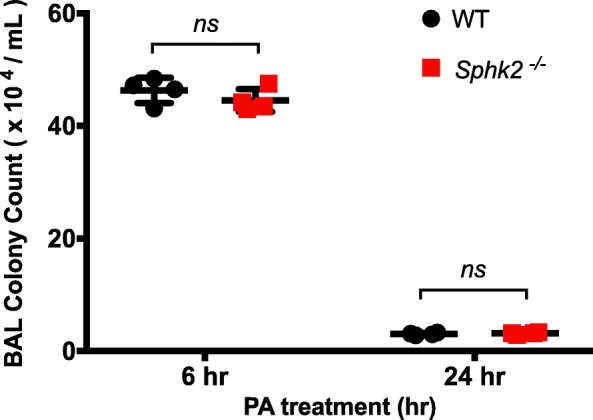
Validation of *Pseudomonas aeruginosa (PA)* inoculum by BAL culture post-inoculation. PA was inoculated into the trachea at a dose of 1 × 10^6^ CFU/mouse and following administration BAL was collected at 6 or 24 h, post-inoculation and plated out on sheep blood agar plates. Bacterial colony count was done after 24 h. We noted that there was no significant difference following administration of PA by intratracheal route as the BAL at 6 or 24 h showed comparable number of bacterial colony count suggesting no difference in the bacterial load inoculated. WT mice showed a mean colony count of 46.3 × 10 ^4^/ml and the *Sphk2* KO 44.55 × 10 ^4^/ml at 6 h post-inoculation and at 24 h WT had a mean colony count of 3.05 × 10 ^4^/ml and *Sphk2* KO, 3.16 × 10 ^4^/ml

Equal dose of live *PA* was administered into the trachea of WT and *Sphk2*^*−/−*^ mice as described in the methods section. BAL was collected and bacterial colony count performed. There was no significant difference in the colony count between WT (mean 46.3 × 10 ^4^/ml) and the *Sphk2* KO (mean 44.55 × 10 ^4^/ml) at 6 h post-inoculation or at 24 h (WT = mean 3.05 × 10 ^4^/ml and *Sphk2* KO = mean 3.16 × 10 ^4^/ml) as shown in Fig. 13.

## Discussion

Activation of innate host defense mechanism represents one of the initial responses by the host organism to an invading pathogen. This is essential for clearance of the pathogen and limit further damage. *PA* is responsible for a variety of infections in the nosocomial setting. It also causes serious pulmonary infections of CF patients, and has been reported to alter genome expression in the host cell beyond inciting a strong innate immune response [49, 50]. Here, we report the genomic host response in WT mice infected with *PA* and the essential role of SPHK2 in the ensuing pathogenesis that is relevant to pneumonia and sepsis. Our gene expression analysis clearly revealed a link between SPHK2 and several key downstream pathways that play a role, either directly or indirectly in *PA* pathogenesis.

One of the earliest attempts to study transcriptional changes following *PA* infection was carried out in A549 cells [49], wherein interferon regulatory factor 1 (IRF-1) was noted to be activated. In the same study, genes encoding transcription factors such as IkB-휶 (NF-kB Inhibitor Alpha-NF-kBIA), and epithelial-specific transcription factor (ESE-1/ELF3) were up-regulated. In addition, several inflammatory response genes, including monocyte chemotactic protein MCP-1 (*CCL2*) and TNF-α-induced protein A20 (*TNFAIP3*) were up-regulated [49]. The *DPH1* gene, whose product catalyzes conversion of histidine residues to diphthamide in elongation factor 2 was also up-regulated. Further, genes involved in a variety of signal transduction pathways that displayed altered expression, such as RhoB GTPase (RHOB), JAK-1 (JAK1), and c-Jun (JUN) were elevated. Our in vivo results are in accordance with the above finding. For instance, expression of *Nfkbia, Nfkb1, Nfkb2, Elf3, Ccl2, Tnfaip3* genes were up-regulated in WT mice treated with *PA* compared to other groups. The *Sphk2*^*−/−*^ *PA* group showed significant degree of protection accompanied by lack of activation of the aforementioned genes belonging to NF-kB activation pathway (Fig. 4). However, no significant changes were noted with respect to the expression of *Dph-1, Rhob, Jak1 or c-Jun* genes in WT *PA* group.

PKC family of isoenzymes are involved in a variety of cellular processes including proliferation, differentiation and secretion. Ichikawa et al also reported PKC family genes to be up-regulated by *PA* in epithelial cells (39). In another study, it has been observed that *PA* activates PKC α to invade middle ear epithelial cells [51]. Activation of PKC α by its phosphorylation in human middle ear epithelial cells (HMEEC) was associated with actin condensation, and blocking the PKC pathway attenuated the ability of bacteria to invade HMEECs and subsequent actin condensation. In our study we noted significant differential regulation of genes in the PKC pathway. A similarity between the two studies was noted in that infection by *PA* in WT mouse caused upregulation of genes promoting inflammation and apoptosis. PKC δ (*Prkcd*) was significantly up-regulated in WT *PA* compared to the rest of the group. Also, upregulation of other members of the PKC pathway such as *Sele*, which promotes adhesion of leucocytes [34], *Lcp2* the product of which mediates T-cell receptor mediated signal transduction [52], *Nr4a1* the product of which upon translocation from nucleus to mitochondria causes apoptosis [35] was observed. However, *PA* infection leads to significant downregulation of genes in the PKC pathway including *Gna11* encoding the protein belonging to the family of guanine nucleotide-binding proteins (G proteins), which function as modulators or transducers in various transmembrane signaling systems and *Prkcz*, encoding protein kinase c Ζ, a member of the PKC family of serine/threonine kinases. In the present study, we also observed downregulation of RAS Guanylyl Releasing Protein 2 gene expression and the corresponding protein activates small GTPases, including RAS and RAP1/RAS3 [53, 54]. In this manuscript we have delved into the genomics of *Sphk2*^*−/−*^ as the mechanism of *PA* infection stimulating phosphorylation of SPHK2 mediated by protein kinase C (PKC) δ and its localization in epithelial cell nucleus has already been described by us [11].

CF patients are highly susceptible to *PA* infection including the chronic colonization and biofilm formation, culminating in pneumonia [55]. In order to understand the pathogenesis of infection and colonization, it is essential to understand the underlying molecular mechanisms, especially at the genomic level. Increased transcription of the mucin genes (*Muc2, Muc5a*) in the respiratory tract in response to the presence of bacteria followed by accumulation of viscous mucus in the airways has been described [56]. However, we did not note any differential regulation in the expression of *Muc2, and Muc5a* genes in our acute murine model of *PA* infection.

Recent studies have shed light on the role played by bacterial pathogens in reprograming host genome by influencing epigenetic factors [57]. Gene modification at epigenetic level holds the potential to alter the response of the host to future infections. *PA* has been shown to induce early T3SS-dependent dephosphorylation and deacetylation of histone H3 in eukaryotic cells. Epigenetic regulation of genes following *PA* infection is an interesting finding of ours in that it can have far reaching implications beyond immediate infection. *PA* infection caused downregulation of Histone1H2bc (*Hist1h2bc*) gene, which encodes a member of the histone 1H2B family. Histone1 families of proteins interact with linker DNA between nucleosomes aiding the compaction of chromatin into higher order structures. This protein, which has antibacterial and antifungal activity, was significantly down-regulated in the WT mice but preserved in the *Sphk2*^*−/−*^. This could contribute to the protection seen in the *Sphk2*^*−/−*^. Downregulation of *Runx1T1* along with that of *Hist1h2bc* is important because Runx1 translocation partner 1 belongs to a family of transcriptional corepressors that interact with both transcription factors bound to promoters of target genes and with histone deacetylases (HDACs). *Sphk2*^*−/−*^ mice infected with *PA* did not reveal any downregulation of the aforementioned genes in the epigenetic pathway. Interestingly, *PA* infection of WT mouse lung inhibited HDAC1/2 activity, and enhanced H3 and H4 histone acetylation, however, genetic deletion of *Sphk2* in mice attenuated *PA* mediated H3 and H4 histone acetylation [11] . Further, S1P generated in the nucleus of lung epithelial cells by SPHK2 after exposure to heat-inactivated *PA* modulated HDAC1/2 activity that was blocked by inhibition of SPHK2 activity with a specific inhibitor, ABC 294640 [11], thus confirming the microarray and PW analysis data.

Activation of the Epithelial Sodium Channel (ENaC) by Alkaline Protease in *PA* treated cells has been reported [58]. Our study did not show any differential regulation of ENaC genes per se but members of the EnaC pathway genes, for example, the epithelial sodium channel Furin and endothelin1 (End 1) were up-regulated in the WT *PA* group. Furin plays an important role in the activation of exotoxin A, which is a major virulence factor of *PA* [59, 60]. Endocytosed exotoxin A is processed by Furin and is transported retrograde to the endoplasmic reticulum. The toxin activates *Pseudomonas* exotoxin A by specific cleavage and inhibits protein synthesis by ADP ribosylation of elongation factor 2, triggering cell death [61]. As noted earlier, WT *PA* group was associated with up-regulation of Furin gene unlike similarly treated *Sphk2*^*−/−*^ mice (Fig. 7). This indicates that *PA*-induced activation of Furin is somehow dependent on *SphK2* gene activity and could be the principal mechanism through which host combats *PA* infection. Further studies are in progress to validate the above hypothesis. We suggest that the biological phenomenon of reduced intensity of *PA* pneumonia in the *Sphk2* KO mice could involve multiple pathways and the significant ones identified in our analyses is presented here.

Members of the sphingolipid family play a significant role in protection against *PA* infection while S1P generated by SPHK2 aggravates the *PA* pneumonia [16–18]. It has been recently demonstrated that glucosylceramide acts in vivo in concert with sphingosine and ceramide to determine the pulmonary defense against *PA* [62].

## Conclusion

This study sheds light on the key role played by SPHK2 in facilitating the *PA* infection in the host and how the ‘hijacking’ of the host genome by the invading organism can be resisted by blocking the SPHK2/S1P pathway. *PA* infection caused significant upregulation of the genes related to NF-κB pathway and members of the PKC family. Also, *PA* infection up-regulated Furin gene, which plays a critical role in the activation of exotoxin A, a major virulence factor of *PA*. This study specifically gives information on the differential expression of genes following *PA* in an in vivo system lacking *Sphk2* gene showing protection against *PA* pneumonia. Further mechanistic studies are required to prove the hypotheses that could be derived from the genomic information provided by this study. Translational application of the SPHK2 pathway has the potential to have far reaching implications in the therapy of *PA* infection in CF patients by two mechanisms such as 1) Targeting the SPHK2 pathway mediated pathology and 2) Reducing the virulence of the organism by resisting its alteration of the host genome. Further understanding of this novel mechanism can be harnessed to overcome the virulence of the organism thus opening up new avenues in the therapy of *PA* infections across various species.

## Supplementary information

**Additional file 1 : Figure S1.** Lungs from WT or *Sphk2*^*−/−*^ mice were removed for protein extraction as described in Materials and Methods. Whole lung homogenates were subjected to SDS-PAGE and Western blotting **(A)**. Immunoblot showed almost absent expression of SPHK2 in the *Sphk2*^*−/−*^ mice compared to the WT mice.

**Additional file 2.** Details of the primers used to perform RTPCR on genes used to validate the RNAseq daa.

**Additional file 3.** A description of top 50 pathways obtained by enrichment as described in the methods. Genes of significance obatined from the pathways were used for analyses and generation of heatmaps.

## Data Availability

The RNAseq datasets supporting the conclusions of this article are available in the National Center for Biotechnology Information Gene Expression Omnibus repository, with unique persistent identifier of NCBI tracking system accession number. The hyperlink to the datasets is given below. https://www.ncbi.nlm.nih.gov/geo/query/acc.cgi?acc=GSE121359

## Abbreviations

ANOVA: Analysis of variance
CF: Cystic fibrosis
CFTR: Cystic fibrosis transmembrane regulator
CFU: Colony-forming units
CFU: Colony-forming units
COPD: Chronic obstructive pulmonary disease
CTRL: Control
DEG: Differentially expressed genes
EA: Enrichment analyses
EGF: Epidermal growth factor
ENaC: Epithelial sodium channels
ESE-1/ELF3: Epithelial-specific transcription factor
HDAC: Histone deacetylases
His: Histone
HMEEC: Human middle ear epithelial cells
IRF-1: Interferon regulatory factor 1
MCP: Monocyte chemotactic protein
Muc: Mucin
PA: *Pseudomonas aeruginosa*
PKC: Protein Kinase C
PW: Pathway
RHOB: RhoB GTPase
RIN: RNA integrity number
RT-PCR: Reverse transcriptase polymerase chain reaction
S1P: Sphingosine-1 phosphate
SPHK: Sphingosine kinases
TNFAIP3: TNF-α-induced protein A20
TNFRSF13B: TNF superfamily member 13b
WT: Wild type
