# Supplementary figures and images for: Genetic deletion of Sphk2 confers protection against Pseudomonas aeruginosa mediated differential expression of genes related to virulent infection and inflammation in mouse lung
Source: BMC Genomics. 2019 Dec 16;20:984. doi: 10.1186/s12864-019-6367-9 (PMC6916461; doi:10.1186/s12864-019-6367-9)

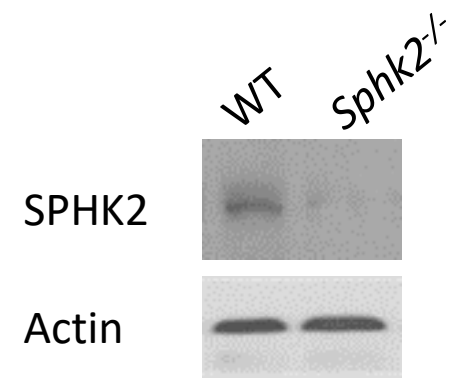

Supplement: Supplementary file 1 — Additional file 1 : Figure S1. Lungs from WT or Sphk2−/− mice were removed for protein extraction as described in Materials and Methods. Whole lung homogenates were subjected to SDS-PAGE and Western blotting (A). Immunoblot showed almost absent expression of SPHK2 in the Sphk2−/− mice compared to the WT mice. [file 12864_2019_6367_MOESM1_ESM.pdf]
