# Supplementary material for: Genetic deletion of Sphk2 confers protection against Pseudomonas aeruginosa mediated differential expression of genes related to virulent infection and inflammation in mouse lung
Source: BMC Genomics. 2019 Dec 16;20:984. doi: 10.1186/s12864-019-6367-9 (PMC6916461; doi:10.1186/s12864-019-6367-9)
Supplement: Supplementary file 2 — Additional file 2. Details of the primers used to perform RTPCR on genes used to validate the RNAseq daa. [file 12864_2019_6367_MOESM2_ESM.docx]

| ***Genes*** | ***Forward Primer*** | ***Reverse Primer*** |
| --- | --- | --- |
| *Spata 13* | 5'-GTTAGGCTTCGAGTCAATCAGG-3' | 5'-ATGACGTTGGTCCGCATCTGC-3' |
| *Kdm6b* | 5'-TGAAGAACGTCAAGTCCATTGTG-3' | 5'-TCCCGCTGTACCTGACAGT-3' |
| *G0S2* | 5'-GTGAAGCTATACGTGCTGGG-3' | 5'-CCGTCTCAACTAGGCCGAG-3' |
| *Fosl2* | 5'-CCAGCAGAAGTTCCGGGTAG-3' | 5'-GTAGGGATGTGAGCGTGGATA-3' |
| *Dgat1* | 5'-TCCGTCCAGGGTGGTAGTG-3' | 5'-TGAACAAAGAATCTTGCAGACGA-3' |
| *Odc1* | 5'-GACGAGTTTGACTGCCACATC-3' | 5'-CGCAACATAGAACGCATCCTT-3' |
| *Smtnl2* | 5'-CACAGCTCGAACGCTTGAC-3' | 5'-AAGGTGGCATGACTGGAGAAG-3' |
| *Sox17* | 5'-GATGCGGGATACGCCAGTG-3' | 5'-CCACCACCTCGCCTTTCAC-3' |
| *Clec1a* | 5'-AAGAGCTGGATTTTGCCATGC-3' | 5'-AGAGCCCTGTCCAATAAGAGTAG-3' |
| *Hdc* | 5'-CGTGAATACTACCGAGCTAGAGG-3' | 5'-ACTCGTTCAATGTCCCCAAAG-3' |

Sequences of SYBR green mouse primers used for RT-PCR.
